# Violation of the transit-time limit toward generation of ultrashort electron bunches with controlled velocity chirp

**DOI:** 10.1038/srep32567

**Published:** 2016-09-22

**Authors:** Seok-Gy Jeon, Dongwon Shin, Min Sup Hur

**Affiliations:** 1 Applied Electromagnetic Wave Research Center, Korea Electrotechnology Research Institute (KERI), Ansan 426-170, Republic of Korea; 2 Department of Physics, UNIST, Ulsan, 44919, Republic of Korea

## Abstract

Various methods to generate ultrashort electron bunches for the ultrafast science evolved from the simple configuration of two-plate vacuum diodes to advanced technologies such as nanotips or photocathodes excited by femtosecond lasers. In a diode either in vacuum or of solid-state, the transit-time limit originating from finite electron mobility has caused spatiotemporal bunch-collapse in ultrafast regime. Here, we show for the first time that abrupt exclusion of transit-phase is a more fundamental origin of the bunch-collapse than the transit-time limit. We found that by significantly extending the cathode-anode gap distance, thereby violating the transit-time limit, the conventional transit-time-related upper frequency barrier in diodes can be removed. Furthermore, we reveal how to control the velocity chirp of bunches leading to ballistic bunch-compression. Demonstration of 0.707 THz-, 46.4 femtosecond-bunches from a 50 μm-wide diode in three-dimensional particle-in-cell simulations shows a way toward simple and compact sources of ultrafast electron bunches for diverse ultrafast sciences.

Generation of ultrashort and ultrafast electron bunches is a subject of great interest, due to their use in terahertz wave generators, ultrafast electron spectroscopy and microscopy, accelerator science, and free electron lasers. The vacuum devices^1^ manipulating electrons in vacuum where electrons have the highest mobility are the most efficient devices of generating ultrashort electron bunches^2^,^3^,^4^,^5^,^6^,^7^,^8^,^9^,^10^. For several decades, advanced vacuum devices have been demonstrated^11^,^12^,^13^, adopting micro-fabricated circuits along with field emission arrays (FEAs)^14^,^15^ or carbon nanotubes^16^,^17^,^18^ replacing thermionic cathodes^19^,^20^,^21^ or tungsten needles^22^. Recently reported nanotips^2^,^3^,^4^ and photocathodes^16^,^23^,^24^ equipped with femtosecond lasers have attracted great interest because they are promising technologies for an ultrafast (or ultrahigh frequency) vacuum diode. However, electronic diodes depending on spatiotemporal transport of electrons (or carriers) in vacuum as well as in solid-state materials still suffer from the transit-time limit (or effect): as the modulation frequency increases, the AC electric field flips its direction before the electrons reach the anode (Fig. 1a). Hence increasing the AC frequency is limited by the transit-time of electrons. Intuitively, the electron bunch-collapse caused by the inverse field can seemingly be prevented by reducing the gap distance (Fig. 1b), thereby reducing the transit-time, which has pushed up the upper frequency barrier (or the cutoff frequency) of typical diodes toward a few GHz.

Here we reveal a more fundamental physical origin of the bunch-collapse which happens when complete transit of electrons fails. Conventionally, the finite electron mobility in high frequency modulation, leading to the transit-time limit, has been pointed out to explain the bunch-collapse. However we discovered that, regardless of the electron mobility, the appearance of the excluded transit-phase (ETP), deduced from our theoretical analysis on the transit-time limit, is a more essential cause of the bunch-collapse. Here ETP is defined by prohibited ranges of transit-phase (equivalently transit-time) at specific frequencies, which appear as periodic discontinuous jumps in the graph of the transit-phase as a function of the AC frequency. Because the prohibited (i.e. excluded) range changes depending on the time of electron emission relative to a given AC period, electrons emitted from the cathode even with a closely adjacent time interval can be widely separated when they reach the anode, leading to an abrupt bunch-collapse at a specific frequency band. The concept of ETP, which is introduced and termed here for the first time as far as we know, is very useful in understanding the conventional upper barrier of AC frequency, and also in finding a condition to break the frequency barrier by suppressing the appearance of ETP. With a biased DC (direct current) electric field determined by our theory, extension of the gap distance (Fig. 1c) significantly violating the conventional transit-time limit can lead to the prevention of bunch-collapse in the entire frequency range. Moreover, we also found that by adjusting the transit-phase of an electron bunch to integer multiples of 2π, the velocity distribution of electrons can be chirped so that ballistic bunch-compression is brought on the electron bunch while it passes through the anode. From three-dimensional particle-in-cell simulations, a micro-bunched train of electrons with ultrashort 46.4 femtosecond bunch-length and 0.707 THz frequency could be obtained from a 50 μm-wide two-plate diode, matching our theoretical prediction.

## Results

### Analysis of the electron bunch-collapse in a diode

**Figure 1:**
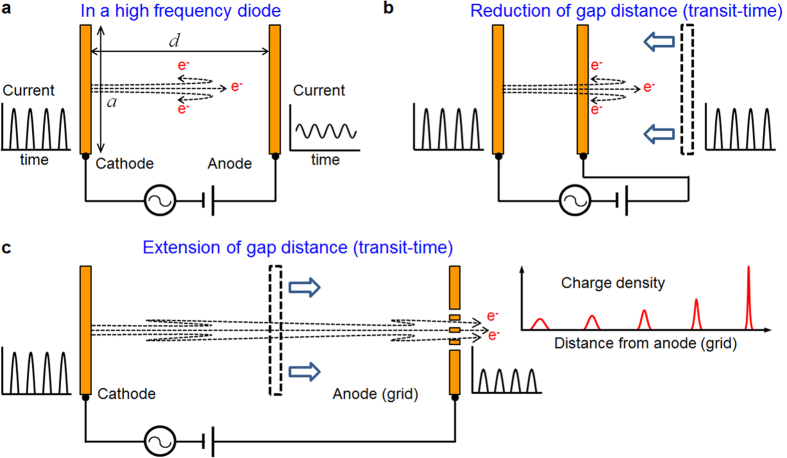
High frequency vacuum diodes. (**a**) Schematic of the DC-biased AC-driven vacuum diode. In the figure, *d* is the gap distance between the two electrodes, and *a* is the side length of the electrode. Bunched electrons are emitted but some electrons drift back toward the cathode by encountering an inverse electric field at high frequency. Consequently, well-separated current pulses at the cathode collapse at the anode (see the current vs time curve beside the cathode and the anode). Note that even in the conventional diode, a small DC field is sometimes used to decrease the activation amplitude of the AC field. (**b**) By reducing the gap distance to minimize the transit-time of electrons, every electron can traverse the gap space preserving the bunched shape (conventional way). **(c)** With a proper DC-bias field, even after significantly extending the gap distance every electron can traverse the gap space preserving the bunched shape. At a certain condition, electron bunches passing through the anode (grid) are compressed during the ballistic motion (proposed way).

Our analysis is performed on one-dimensional (1-D) motion of electrons along *z*-direction across the cathode and the anode (Fig. 1). For the 1-D analysis, a sufficiently small cathode area is assumed. Moreover thermal spread of electrons inherently given at the moment of emission and the space charge force between electrons are neglected in the following analytical calculations. Those effects are considered in the particle-in-cell (PIC) simulations presented later. When an electron is emitted at time *t*_0_, after *t* from the emission, the phase of the AC field seen by the electron is *ω* (*t* + *t*_0_) = *ωt* + *ϕ*. Here *ϕ* = *ωt*_0_ is the initial phase of the electron at the moment of emission relative to the AC field. Thus the electric force exerting on each electron is, from a *z*-directional AC electric field in parallel with a DC-bias field, written by:


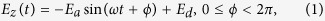


where *E*_*a*_, *E*_*d*_, *ω* and *ϕ* represent amplitude of the AC field, magnitude of the DC-bias field, angular frequency (2*πf* ), and initial phase of the AC field, respectively. Note that time *t* is referenced to each electron so that an electron sees an electric field −*E*_*a*_ sin *ϕ* + E_*d*_ at the moment of emission. Without loss of generality, we confine *E*_*a*_ to be positive. Though our analysis is valid for both thermionic and field emission cathodes in principle, here we consider the carbon nanotubes (CNTs) field emitters as an example of a cathode material. The benefits of using the field emission cathode are the absence of thermionic heaters and the relatively high nonlinearity in the relation between emission current density and applied electric field, which enable manufacture of compact and high frequency vacuum devices. The current density by field emission from CNTs cathodes obeys the well-known Fowler-Nordheim (FN) formula.


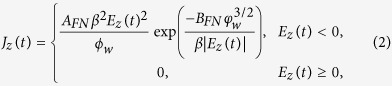


where *β* is the field enhancement factor, *ϕ*_*w*_ is the work function, and *A*_*FN*_ and *B*_*FN*_ are 1.56 × 10^6^AV^−2^ eV and 6.83 × 10^6^AV^−1^ eV, respectively^18^.

To make the further analysis simple, we assume that every electron is emitted with zero velocity at the cathode surface (*z* = 0). The maximum current density is acquired when the electric field is strongest in the negative *z*-direction, i. e. *E*_*z*_(*t*) = −*E*_*a*_ + *E*_*d*_, which is defined by −*E*_*M*_ (*E*_*M*_ > 0). To prevent material breakdown, *E*_*M*_ should be kept smaller than the breakdown threshold of CNTs. We integrated the equation of motion with a normalized DC-bias field defined by 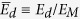 which has a confined range 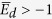 from the definition of *E*_*M*_, leading to the velocity and position of an electron as:


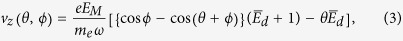



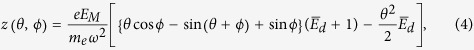


where *ωt* is replaced by the transit-phase *θ*, and the mass and charge of an electron are denoted by *m*_*e*_ and *e*, respectively. The transit-phase (or equivalently the transit-time) of an electron emitted with *ϕ* is a function of AC frequency *ω*. By equating *z*(*θ*, *ϕ*) = *d* in Eq. (4), we can obtain the relation between *θ* and *ω*:


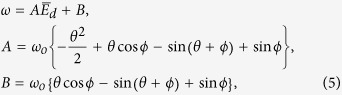


where 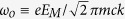, *c* is the speed of light and the dimensional aspect ratio *k* is defined as *d*/*a*. The parameter *ω*_*o*_ is introduced to normalize *ω*. Physically *ω*_0_ corresponds to the approximate maximum frequency limit in a vacuum diode when the DC-bias is absent. When there exists a real positive set of (*θ*, *ω*) (*θ* > 0, *ω* > 0) satisfying Eq. (5), that electron can traverse completely the gap distance and escape the system. Here, though our analysis is for 1-D systems, we introduce transverse dimension *a*, which is perpendicular to the direction of distance *d*. Then the hexahedral space with volume *a*^2^*b* enveloped by conducting sidewalls can be a cavity resonator which contains various electromagnetic resonance modes. One of them is the TM_110_ mode and its resonance frequency, described by 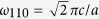, is matched with *ω* in equation (1). Then, because the AC electric field of the TM_110_ mode has only a *z*-directional component, the electric field inside the cavity can be adjusted to match equation (1) with an additional DC-bias field^22^. Consequently our analysis is valid for a cavity resonator diode as well as a simple two-plate diode. Here it is worthwhile to note that a strong AC electric field can be resonantly accumulated in a cavity resonator. To apply a strong DC-bias to a resonator preserving a high quality-factor in a practical system, a two-dimensional (2-D) slab of photonic crystal (PC) made of dielectric materials^25^,^26^,^27^ can be used for the resonator. The 2-D PC slab sandwiched by two conducting plates can support the TM_mn0_ electromagnetic modes^26^,^27^, while a high DC voltage can be applied between the plates separated by the insulating dielectrics.

**Figure 2:**
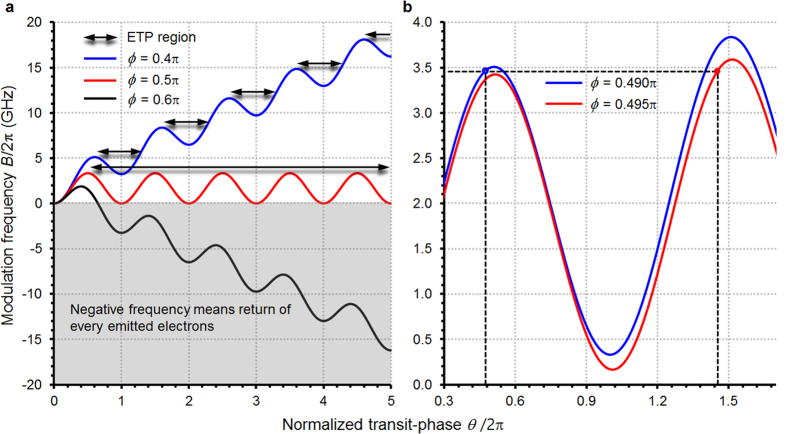
Correlation between modulation frequency and transit phase for zero DC-bias field. (**a**) Modulation frequency *B*/2*π* of a vacuum diode corresponding to normalized transit phase *θ*/2*π* is given with different initial phase *ϕ* of AC electric field which electrons see at their emission. Half of the emitted electrons encounter a GHz level of upper frequency barrier because negative frequency means the electrons return to the cathode. For the other half of the electrons, the appearance of the excluded transit-phase (ETP) regions makes the transit phase discontinuous, leading to failure of the diode. (**b**) The ETPs make an abrupt and discontinuous expansion of the transit-phase even for electrons emitted with closely adjacent initial phases *ϕ* = 0.490*π* and *ϕ* = 0.495*π*.

We first address 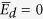 case (zero DC-bias). Around *ϕ* = *π*/2 where the electron emission is most abundant, the modulation frequency *B*/2*π* given by equation (5) as a function of *θ* is shown in Fig. 2a. In the following, as typical values, we used *E*_*M*_ = 2.0 /μmV and *k* = 0.025, which yield *ω*_*o*_ = 1.05 × 10^10^ (rad/s). One noticeable feature in the figure is that there is an intrinsic upper frequency barrier. When the modulation frequency is larger than about 3.5 GHz in Fig. 2a, the transit-phase of electrons emitted with 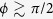 does not have roots of equation (5), implying that half of the electrons fail to arrive at the anode. The other half emitted with 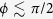 can have transit-phases satisfying equation (5) for arbitrarily high frequencies. However, in that case, the *excluded transit-phase* (ETP) regions come out discretely and periodically (Fig. 2a); for example, when the frequency is about 3.45 GHz in Fig. 2b, the transit-phase of an electron emitted with *ϕ* = 0.490*π* is determined by the first crossing point located at *θ*/2*π* = 0.47. The second and the third crossing points are returning-phases to the anode from outside of the gap space, which is meaningless in real diodes since there is no returning electric field outside the gap space. Meanwhile, an electron emitted slightly later (*ϕ* = 0.495*π*) has the first crossing point at a significantly later transit-phase *θ*/2*π* = 1.44. This means that electrons emitted from the cathode even within a closely adjacent time interval can be considerably separated (bunch-collapse) inside the gap space. Consequently, for the 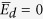 case, the frequency should be low enough to keep the transit-phases of all electrons below the lowest ETP region to prevent the electron bunch-collapse by the transit-phase discontinuity. In the particular case of Fig. 2, the upper limit is at around 3.5 GHz, matching well the previous theoretical and experimental result^13^,^14^.

### Correlation between the modulation frequency and the transit-phase

In the case of vacuum diodes with a DC-biased AC electric field, we can find a general condition to entirely suppress the appearance of ETP for any value of *θ* and *ϕ* by solving ∂*ω*/∂*θ* ≥ 0, which yields 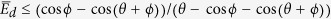, leading to 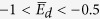. Even for 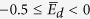, as *θ* increases, *ω* eventually becomes a monotonically increasing function due to the dominant −0.5*θ*^2^ in equation (5). This indicates that, if *ω* is sufficiently high, we can always find a physically meaningful pair of *θ* and *ϕ*, which ensures that every electron eventually escape the gap space. From here, the range of 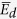 is confined to 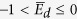 because the positive 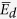 case is qualitatively similar to the 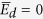 case.

**Figure 3:**
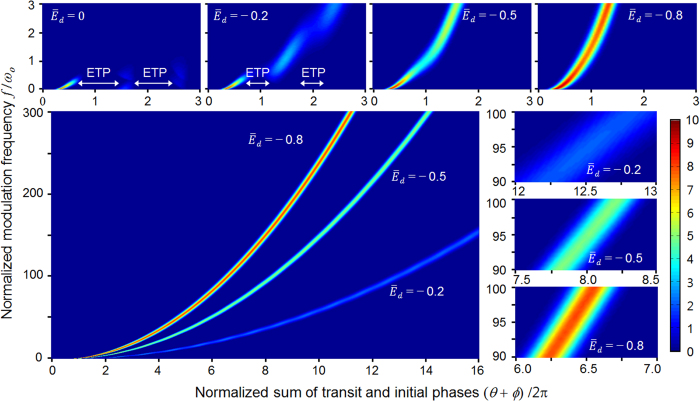
Correlation between modulation frequency and transit-phase. The transit-phase *θ* is numerically calculated for thousands of sample electrons, varying the initial phase *ϕ* of the AC electric field and the modulation frequency *f* normalized by *ω*_*o*_ = 1.05 × 10^10^ (rad/s) for different normalized DC-bias field 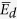. The horizontal width of strips corresponds to the bunch-length and the normalized current density is scaled by color. When 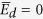 and 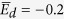, the diodes fail above an upper frequency barrier or at the discrete ETP regions. Otherwise, electrons behave as a well-confined bunch regardless of the modulation frequency, which implies generation of ultrashort electron micro-bunch trains.

Now the most critical concern is whether the electrons behave collectively as a spatiotemporally confined bunch for a large transit-phase prohibited by the transit-time limit. To clarify that, the correlations between the modulation frequency and the transit-phase for different values of 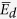 are figured out (Fig. 3) by numerically calculating *θ* for thousands of sample electrons, varying *ω* and *ϕ*. The *x*-coordinate represents the sum of *θ* and *ϕ* normalized by 2*π* so that the *x*-directional width of the strips colored for relative current density representation corresponds to the temporal bunch-length normalized by one AC period. The *y*-coordinate represents the modulation frequency *f* normalized by *ω*_*o*_ = 1.05 × 10^10^ (rad/s) given in the previous section. When the DC-bias field is zero (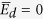), *f*/*ω*_*o*_ is bounded strictly under 1.0 matching to a few GHz as predicted in the previous section. For the 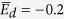 case, when *f*/*ω*_*o*_ is chosen to be around 0.5 or 2.0, an abrupt and considerable expansion of the bunch-length turns up by encountering the ETP regions making a discontinuity in the strips. When 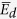 is −0.8 belonging to 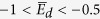, the discontinuity in the strips entirely disappears along with the increase of current density by virtue of the DC-bias enhancement.

Here the most interesting feature is that the bunch-length remains quite bounded even if the modulation frequency enters far into the THz frequency regime which has been forbidden in conventional diodes. A theoretical condition for such spatiotemporal confinement regardless of the modulation frequency is given in the next section. Meanwhile, the reduction of gap distance to satisfy the conventional transit-time limit is seemingly effective for any frequency regime because the first ETP region always appears around the transit-phase of 2*π*. However, it should be noted that as the frequency increases, the dimensional aspect ratio *k* defined by *d*/*a* goes to zero for the transit-phase bounded under 2*π* because the orthogonal distances *a* and *d* are proportional to *f*^−1^ and *f *^−2^, respectively. Thus, the 3-D space configured by *a* and *d* is deformed into a 2-D-like space (an extremely thin 3-D sheet) which causes crucial issues of technology in practical applications. Here, we claim that the suppression of ETP by considerably extending the gap distance accompanied with a selected DC-bias field is a more effective way for an ultrafast vacuum diode.

### Ballistic bunch-compression by controlled chirp in velocity distribution

**Figure 4:**
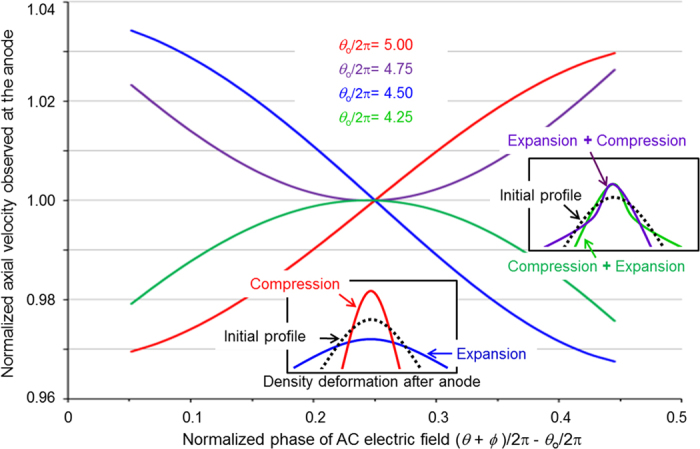
Velocity distribution at the anode. Axial velocity distribution of electrons observed at the anode as a function of the phases of the electrons at the moment they reach the anode. Note that the phase *θ* + *ϕ* on the x-axis corresponds to the time of arrival at the anode multiplied by *ω*. The centers of four distribution curves correspond to electrons emitted with initial phase *ϕ* = *π*/2. The transit-phases *θ*_0_ of those central electrons are 4.25 × 2*π* (green), 4.50 × 2*π* (blue), 4.75 × 2*π* (purple), and 5.00 × 2*π* (red), respectively. The velocity in the y-axis is normalized by the velocity of the centered electrons. By subtracting *θ*_0_/2*π* in x-axis, the centers of the velocity distribution curves are adjusted to meet at the same point.

When the transit-phase is a small fraction of 2π according to the transit-time limit, the electrons reaching the anode earlier have larger *z*-directional velocities. That is one major reason why electron bunches eventually collapse during the drift motion after passing through the anode. However, when the diode configuration is adjusted so that the transit-phase *θ* of an electron emitted with *ϕ* = *π*/2 is 2*πn* (an integer multiple of 2*π*), electrons emitted prior to that (*ϕ* < *π*/2) arrive at the anode earlier and have lower velocities than those emitted later (*ϕ* > *π*/2). Then the electron bunch possessing the chirped velocity distribution will be compressed as the rear part of each bunch catches up to the front part. For the 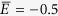 case (Fig. 4), the velocity distributions at the anode, which are normalized by the velocity of electrons with *ϕ* = *π*/2, are numerically calculated as functions of *θ* + *ϕ* (divided by 2*π*) at the moment the electrons reach the anode. Since the phase *θ* + *ϕ* = *ω* (*t* + *t*_0_) is the time of arrival at the anode (multiplied by *ω*), Fig. 4 represents the velocity chirp of electron bunches leaving the anode. It is found that the velocity chirp is determined by the central transit-phase *θ*_0_, which is the transit-phase of the electron emitted with *ϕ* = *π*/2. As will be shown, *θ*_0_ can be controlled by adjusting the diode configuration, in particular, the gap distance. From the positive slope for the 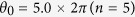 curve, ballistic bunch-compression is expected and it will make an infinitesimally small bunch-length because of the high linearity at the center region taken by the majority of electrons.

From equations (3) and (4), the slope of the velocity distribution curve (Fig. 4) can be estimated as follows. Detailed derivation of equations (6) and (8) can be found in the method section.


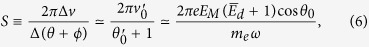


where subscript 0 means evaluations for *ϕ* = *π*/2, Δ stands for variations by difference of *ϕ*_±_ = *π*/2 ± *δ*, the derivatives are with respect to *ϕ* and sufficiently large *θ* and linear variations by Δ are assumed. The most effective bunch-compression occurs when the chirped velocity distribution has the positive maximum slope at *θ*_0_ = 2*nπ*, so that, by setting *z*(*θ*_0_, *π*/2) = *d* in equation (4), the condition to bring on the ballistic bunch-compression is deduced as:


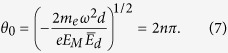


**Figure 5:**
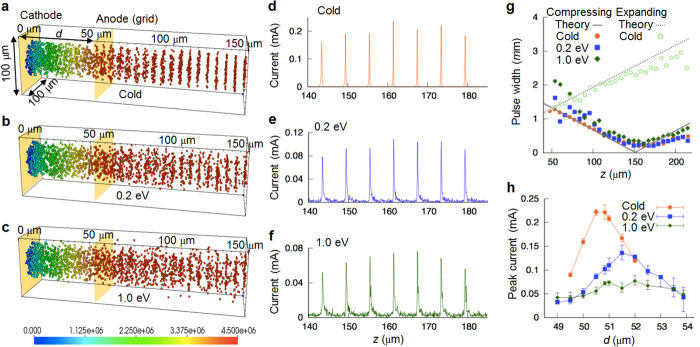
3-D PIC simulations of electron bunching. (**a–c**) Generation of electron bunches and ballistic-compression by controlled velocity chirp for electron temperatures of 0, 0.2 and 1 eV, respectively. (**d–f**) Corresponding currents as functions of position. (**g**) Bunch-length evolution as a function of distance for *d* = 50.84 *μ*m and 53.88 *μ*m, which are the most compressing and expanding cases, respectively. Points are from PIC simulations and the lines are from theory. (**h**) Measurement of peak current at the maximum compression obtained from PIC simulations for cold and thermal cases.

The bunch-length *W* as a function of propagation distance *L* measured from the anode is evaluated by adding the variation of the bunch-length 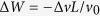, to the bunch-length at the anode *W*_0_, which yields:


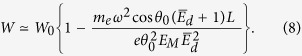


where *W*_0_ is 2*δθ*_0_*eE*_*M*_/*m*_*e*_*ω*^2^. For a sufficiently small *δ*, the velocity distribution is linear as shown at the central region of the curves in Fig. 4 for the compression (*θ*_0_ = 2*nπ*) and expansion (*θ*_0_ = (2*n* + 1)*π*) cases. Since the majority of electrons are concentrated near the central region, the compressing and expanding cases can be estimated fairly accurately by Eq. (8). Here 2*δ* is the bunch-length at the cathode in the phase space originating from *ϕ*_±_ = *π*/2 ± *δ*. By calculating *ωW*_0_/*v*_0_, the bunch-length at the anode is re-evaluated as 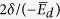 in the phase space so that a constraint 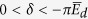 should be followed for the spatiotemporal separation of electron bunches at the anode. Then the bunch-length will become infinitesimally small during the ballistic drift because equation (8) can mathematically be zero. Such bunch-compression into an infinitesimal length is a unique feature distinguished from ballistic bunch-compression in other systems. If *L*_0_ is the distance for the maximum bunch compression for *θ*_0_ = 2*nπ* (maximum-bunching case), equation (8) can be rewritten as 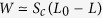 for 0 ≤ *L* ≤ *L*_0_ and 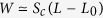 for *L*_0_ ≤ *L*, where 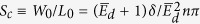. The case of *L*_0_ ≤ *L* can be used to describe the re-expansion of the maximum-compressed bunches. Note that a larger *n* makes both processes of compression and decompression slower, so the longitudinal region where the electron bunches stay compressed becomes longer. From Eq. (8), we can write 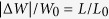, where *L*_0_ can be obtained from *W* = 0 in Eq. (8). For given parameters under consideration, 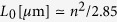. For gap distance larger than 50 μm, *n* = 17 for which L_0_ ∼ 100 *μ*m. Thus the distance where the bunch length is kept under a half of *W*_0_, i.e. the ‘hold-distance’ is more than 100 *μ*m, which is consistent with the 3-D PIC simulations shown later (see Fig. 5g).

### Three-dimensional particle-in-cell simulations

To secure the feasibility of the ultrashort electron micro-bunch generation under more realistic conditions, we conducted 3-D PIC simulations. The simulation parameters are arranged so that the electron emission is gated with a 707 GHz frequency by setting *a* = 300 μm. The gap distance *d* is varied from 49 μm to 54 μm with constant values of 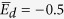 and *E*_*M*_ = 2.0 V/μm. The mesh size is 5 *μ*m in every direction, and the simulation time step is 9.5 × 10^−15^ sec. For the driving AC and DC electric fields, we implemented the analytic TM_110_ mode field and a constant DC electric field into the simulation code. The electric field from the space charge effect, which is neglected in the previous calculations, is now self-consistently calculated in the PIC code. Additionally, the thermal effects of electron emission at the cathode are included. The simulations were conducted with three different temperatures: 0, 0.2, and 1 eV. The temperature of electrons emitted from CNTs is known to be generally of order 1 eV or less^28^. Under these conditions, with *d* = 50.84 *μ*m corresponding to *n* = 17 in equation (7), micro-bunch trains of electrons are clearly observed (Fig. 5a–c). As expected from the theory, the ballistic bunch-compression by the artificially chirped velocity distribution makes electrons in each bunch get together within a sub-micron distance. Corresponding beam currents have remarkably high peak values up to 0.25 mA in the cold case (Fig. 5d), and of order 0.1 mA in the thermal cases (Fig. 5e,f). For this current level, the space charge effect was barely observed. In the thermal cases, the noise current exists between the peaks. We compared the ballistic evolution of the bunch-length calculated from our theory with that from the PIC simulations (Fig. 5g,h). The cases of a cold beam with gap distances 50.84 μm and 53.88 μm, which are theoretically the most compressing and expanding cases, respectively, show good coincidence with the theory (Fig. 5g). For the thermal beams, electrons in each bunch are also compressed though the peak current is lower. The peak current at the maximum compression as a function of the gap distance *d* is presented (Fig. 5h). In the cold case, the peak current appears exactly at the theoretically expected gap distance for the most effective bunch-compression, while the thermal cases tend to shift it up. Interestingly, it is also found that as temperature increases, the dependence of the peak current on the gap distance is weakened, implying that the ballistic bunch-compression is an inherent feature of the vacuum diodes involving a significantly extended gap space.

In summary, we revealed theoretically that the appearance of the ETP region is a fundamental origin of the electron bunch-collapse in high frequency vacuum diodes. From the analysis, contrary to the conventional method for satisfying the transit-time limit by reducing the gap distance in a vacuum diode, we have shown that the suppression of ETP by significantly extending the gap distance with a proper DC-bias electric field is more effective for ultrafast operation of a vacuum diode. In 3-D PIC simulations, a spatiotemporally well-localized ultrashort micro-bunch train of electrons with 1.41 picosecond periodicity (0.707 THz), 46.4 femtosecond bunch-length and 0.014 c mean velocity could be extracted from a 50 μm-wide two-plate diode demanding a transit-phase of 17 × 2π, which is apparently prohibited by the conventional transit-time limit. The simulation result verifies our theoretical prediction that the bunch-length of electron bunches from vacuum diodes can be made ultrashort in the THz frequency regimes by violating the transit-time limit.

## Method

### Derivation of equations (6) and (8)

Since the phase term in equation (1) is arranged so that each electron emits at *t* = 0 measured by its own clock, the initial phase *ϕ* observed by an electron corresponds to the emission time (=*ϕ*/*ω*) of the electron. Hence a larger *ϕ* means later emission time, and vice versa. The maximum emission of electrons at the cathode is set at *ϕ* = *π*/2, which is denoted by the subscript 0 for evaluations with the phase. As the initial phase *ϕ* determines the transit-phase *θ* and the velocity *v*(*θ*, *ϕ*) at the anode, the velocity distribution can be estimated by calculating ∂*v*_0_/∂*ϕ* with an assumption of a sufficiently large *θ*. At the anode, the velocity of an electron emitted with *ϕ* = *π*/2 is, from equation (3):
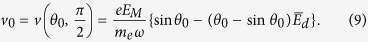


Here *θ*_0_ is the transit-phase for *ϕ* = *π*/2, which can be obtained from equation (4) by setting *z*(*θ*_0_, *π*/2) = *d*:


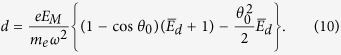


Differentiation of equations (9) and (10) gives:
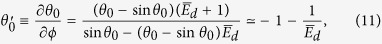



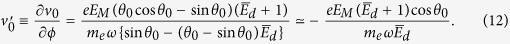
Equation (11) can be used for estimation of the bunch-length just after the anode. An electron reaches the anode at *t* = (*θ* + *ϕ*)/*ω*. When the initial phase at the moment of emission spans *ϕ*_±_ = *π*/2 ± *δ*, the bunch-length at the anode is 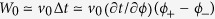, leading to:
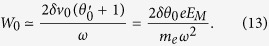


The slope of the velocity distribution in the phase space observed at the anode, i.e. equation (6) in the main text, is obtained from equations (11) and (12). Here the relations 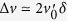 and 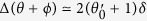 are applied with an assumption that the variations, Δ, for the difference of *ϕ*_±_ = *π*/2 ± *δ* are linear. The assumption of linear variation is reasonable when *θ*_0_ is 2*nπ* or (2*n* + 1)*π*, which follows from equations (11) and (12). The slope has the positive maximum at *θ*_0_ = 2*nπ* which induces the ballistic-compression most effectively. The bunch-length *W* after propagation by length *L* beyond the anode is *W*_0_ + Δ*W*, where 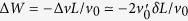. Using equation (12) and (13):
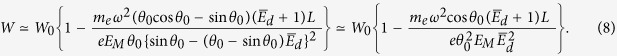


## Additional Information

**How to cite this article**: Jeon, S.-G. *et al*. Violation of the transit-time limit toward generation of ultrashort electron bunches with controlled velocity chirp. *Sci. Rep.* **6**, 32567; doi: 10.1038/srep32567 (2016).

## Supplementary Material

Supplementary Information
