# Supplementary material for: Violation of the transit-time limit toward generation of ultrashort electron bunches with controlled velocity chirp
Source: Sci Rep. 2016 Sep 22;6:32567. doi: 10.1038/srep32567 (PMC5032026; doi:10.1038/srep32567)
Supplement: Supplementary Information [file srep32567-s1.pdf]

nature publishing group npg

In accordance to Nature Publishing Groups Authorship Policy we agree to change the authors of the manuscript as indicated below.

**NAME OF JOURNAL:** Scientific Reports

**TITLE OF MANUSCRIPT:** Violation of the transit-time limit toward generation of ultrashort electron bunches with controlled velocity chirp

**MANUSCRIPT NUMBER:** SREP-16-18962A

**CORRESPONDING AUTHORS NAME: Min Sup Hur**

PREVIOUS AUTHOR NAMES:
Seok-Gy Jeon, Min Sup Hur, Dongwon Shin

UPDATED AUTHOR NAMES:
Seok-Gy Jeon, Dongwon Shin, Min Sup Hur

**CHANGE TO AUTHOR LIST: We placed the corresponding author at the last author,following the general convention in author listing.**

| Print Name | Signature | Date |
|---|---|---|
| Dongwon Shin | | July. 15. 2016 |
| Seok-Gy Jeon | | July 15. 2016 |
| Min Sup HUR | | July 15. 2016 |
| | | |
| | | |
| | | |
| | | |

15961150 / [illegible] / 2016-07-15 11:52 SEMES Confidential
